# Complete Disappearance of Choroidal Metastasis from Lung Adenocarcinoma Treated with Bevacizumab and Chemotherapy

**DOI:** 10.1155/2015/142408

**Published:** 2015-05-07

**Authors:** Hampig Raphael Kourie, Joelle Antoun, Alexandre Schakal, Elie Nasr, Marwan Sahyoun, Joseph Kattan

**Affiliations:** ^1^Department of Hematology-Oncology, Faculty of Medicine, Saint Joseph University, Damas Street, P.O. Box 17-5208, Beirut, Lebanon; ^2^Department of Ophthalmology, Faculty of Medicine, Saint Joseph University, Damas Street, P.O. Box 17-5208, Beirut, Lebanon; ^3^Department of Radiotherapy, Faculty of Medicine, Saint Joseph University, Damas Street, P.O. Box 17-5208, Beirut, Lebanon; ^4^Department of Ophthalmology, Eye & Ear Hospital, Naccash Road, Mar Mansour Street, P.O. Box 70-933 Dbayeh, Beirut, Lebanon

## Abstract

Choroidal metastasis from lung cancer is uncommon. We report a case of choroidal metastasis as an inaugural manifestation of lung adenocarcinoma, successfully treated by docetaxel, cisplatinum, and intravenous bevacizumab as an antiangiogenesis therapy. A complete remission was obtained after 4 cycles and maintained after six cycles. This case report demonstrates the importance of the systemic bevacizumab and chemotherapy in the treatment of choroidal metastasis from adenocarcinoma of the lung.

## 1. Introduction

Metastatic tumors to the choroid are the most common intraocular malignancies [1, 2]. The lung represents the first primary site for choroidal metastasis among men and the second among women after the breast [1–3]. Decrease in visual acuity or other ophthalmic manifestations as the initial clinical presentation of lung cancer primarily is infrequent [3].

Available treatment options are external beam radiotherapy and plaque radiotherapy, while new methods like surgical resection, transpupillary thermotherapy, and intravitreal chemotherapy offer promises for the future [2]. The use of systemic chemotherapy alone or with targeted therapy for choroidal metastases from a primary lung cancer is not widely evaluated. We report a case of choroidal metastasis as an inaugural manifestation of lung cancer, successfully treated by chemotherapy and systemic bevacizumab as an antiangiogenesis therapy.

## 2. Case Presentation

**Figure 1:**
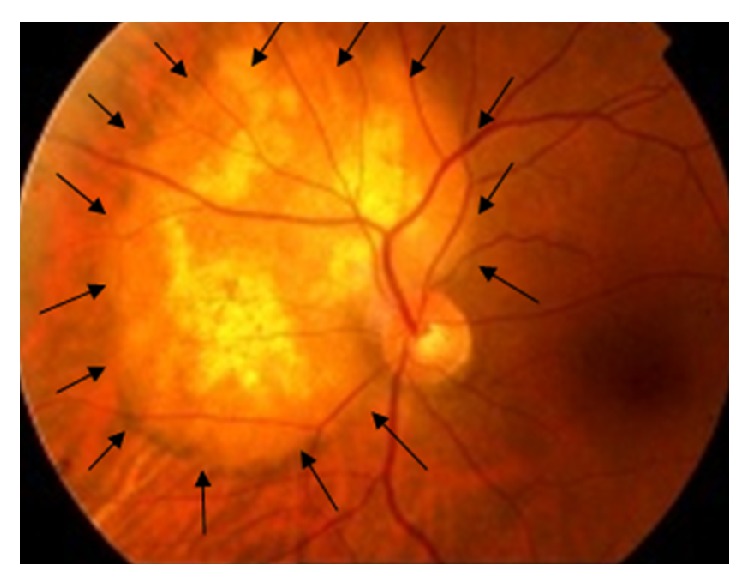
Fundus photo revealed the presence of a choroidal yellow-white elevated lesion, superior-nasal to the disc, with an orange pigmentation on its surface.

MS is a 67-year-old nonsmoker woman having in her past medical history well controlled diabetes and hypothyroidism. She is followed up for a chronic glaucoma in both eyes. In February 2013, she presented with a blurred vision in the left eye for the last two months. Her best-corrected visual acuity (BCVA) was 20/20 in both eyes. The slit lamp examination was unremarkable. Her pupils were of normal size and reactive to light. Her intraocular pressure was within normal limits. Her ocular movements in all gazes were normal. Her fundus exam revealed in the left eye a choroidal yellow-white elevated lesion, nasal to the optic nerve, with an orange pigmentation on its surface (Figure 1). The lesion had basal dimensions of 9∗6 mm.

**Figure 2:**
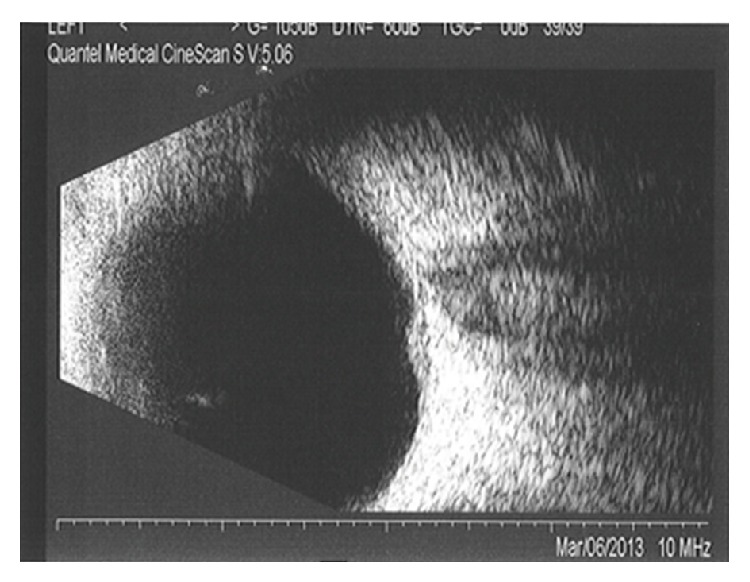
At diagnosis, B-scan ultrasound showed an elevated choroidal lesion with moderate internal reflectivity in the left eye.

**Figure 3:**
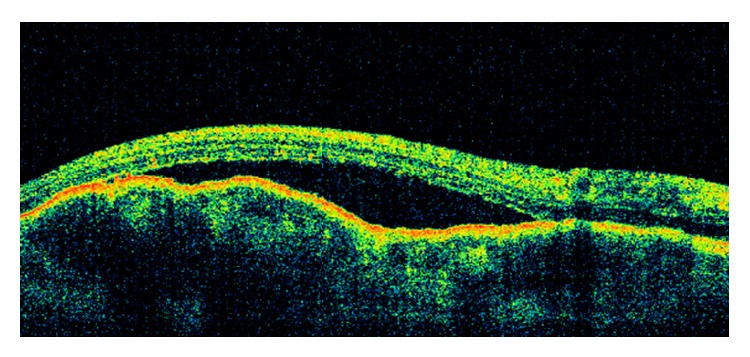
At diagnosis, optical coherence tomography of the left eye demonstrated a dome-shaped elevation of both the neurosensory retina and retinal pigment epithelium- (RPE-) Bruch reflectivity associated with subretinal fluid.

Her fluorescein angiography (FA) showed hypofluorescence on the early phase, followed by hyperfluorescence with leakage on the late phase, associated with pinpoints on the surface. A B-scan ultrasound revealed a dome-shaped elevated choroidal lesion with moderate internal reflectivity of 1.5 mm height (Figure 2). Spectral-domain optical coherence tomography (SD-OCT) demonstrated a dome-shaped elevation of both the neurosensory retina and retinal pigment epithelium- (RPE-) Bruch reflectivity associated with subretinal fluid, in addition to a thickening of the RPE-Bruch reflectivity overlying the choroidal elevation in the left eye (Figure 3).

The patient was referred for a suspicious metastatic choroidal lesion in the left eye. Brain, orbital, and total body metabolic MRI showed a left choroidal lesion and an apical left suspicious primary pulmonary lesion of 12 mm. This unique pulmonary lesion was confirmed by chest CT scanner which showed also multiple infracentimetric mediastinal and hilar lymph nodes. A pulmonary CT-guided biopsy showed moderately differentiated adenocarcinoma. A clinical diagnosis of choroidal metastasis from a pulmonary adenocarcinoma was made.

**Figure 4:**
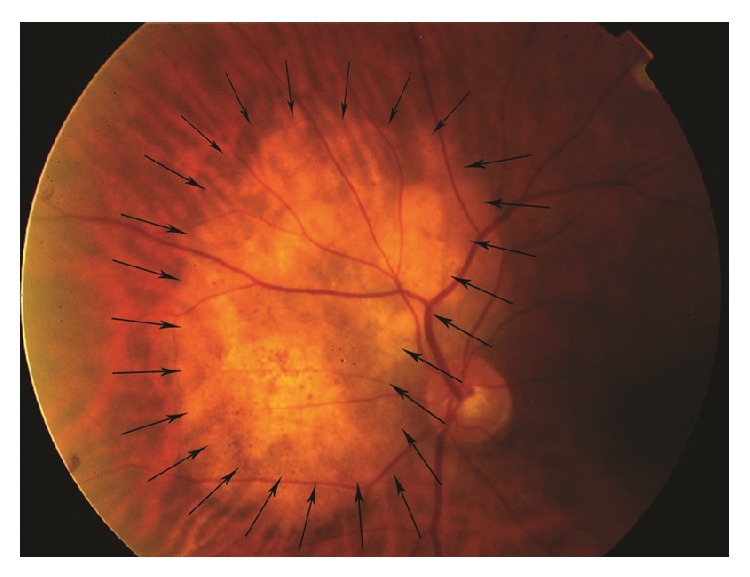
Fundus photo showed a decrease in the height of the choroidal lesion after 4 cycles of chemotherapy and intravenous bevacizumab.

**Figure 5:**
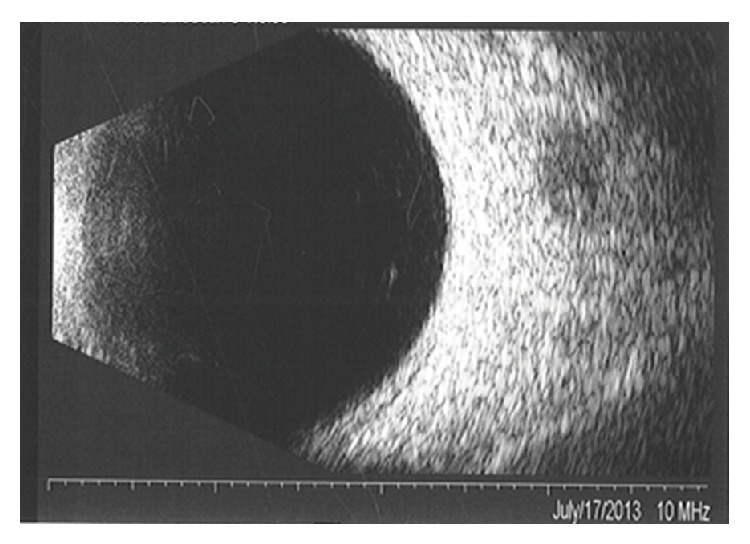
B-scan ultrasound showed a complete regression of the choroidal lesion after 4 cycles of chemotherapy and intravenous bevacizumab.

**Figure 6:**
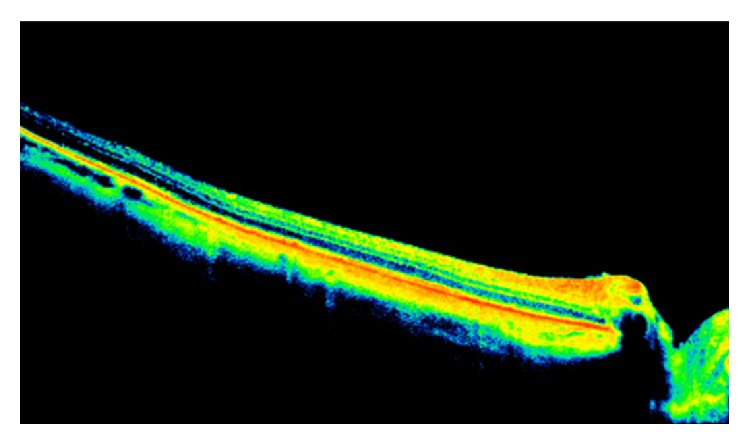
Optical coherence tomography of the left eye showed a complete resolution of the subretinal fluid after 4 cycles of chemotherapy and intravenous bevacizumab.

Doublet chemotherapy including docetaxel and cisplatinum was started along with bevacizumab 10 mg/kg every 3 weeks. After 4 cycles, lung primary cancer decreased to 9 mm and ophthalmologic examination showed complete regression of the choroidal lesion and complete resolution of the fluid, as demonstrated by the B-scan and the SD-OCT (Figures 4, 5, and 6).

At the end of 6 cycles, chest scan showed a remaining 9 mm nodule and the ophthalmology workup a complete disappearance of the choroid lesion. The patient did not experience any systemic or ocular toxicity. A final PET CT-scan done was normal beside the residual pulmonary lesion. The patient will receive irradiation of the left choroid lesion bed (40 Gy in 20 sessions) followed by surgical resection of the residual lung disease.

## 3. Discussion

The choroid is a major site for the development of metastasis within the eye due to its abundant arterial supply and its favorable microenvironment for the seeding of cancer cells. According to the literature, the incidence of ocular metastases from lung cancer is about 2 to 7% [4, 5]. In most cases (66–97%), the diagnosis of a systemic cancer is established before the detection of choroidal metastasis [6]. In contrast to those with breast cancer, patients with lung cancer generally have poorer outcomes and lower survival rates. This is believed to be responsible for a much lower percentage of choroidal metastasis being related to lung cancer [7]. Thus, symptomatic choroidal metastasis as the initial presentation of lung cancer is an infrequent occurrence and is confined to reports of single cases or small series [8].

Differential diagnosis of choroidal metastasis includes amelanotic choroidal melanoma, choroidal osteoma, choroidal hemangioma, posterior scleritis, and other rare lesions. Metastatic tumors usually have a creamy yellow appearance. On fluorescein angiography, they are hypofluorescent in the early phases and become progressively hyperfluorescent in the late phases [9]. B-scan ultrasound shows an echogenic subretinal mass with diffuse, ill-defined borders. Overlying retinal detachment is common and sound attenuation in the lesion is usually moderate [10]. OCT has provided additional useful information in the evaluation of the retinal pigment epithelium (RPE) and retina. Many features are characteristic of choroidal metastasis such as the presence of subretinal fluid and the marked irregularity of the RPE with thickening and gross undulation [11].

Choroidal metastases are usually treated in a palliative way with radiotherapy, enucleation, or transpapillary thermotherapy. Actually, few reported cases showed partial or complete response of the choroidal metastases to chemotherapy alone or chemotherapy associated with targeted therapy. Despite limited published data regarding its use, bevacizumab, the monoclonal antibody against the vascular endothelial growth factor (VEGF), is a promising new therapeutic option when used systemically [12] or intravitreally [13–15] and may be an acceptable alternative to the conventional modalities.

These reports demonstrated antiangiogenic and antipermeability effects of bevacizumab on the new tumor vessels. This forms the rationale for the use of systemic bevacizumab in our patient, apart from the fact that a platinum doublet in combination with bevacizumab is an approved therapy for lung adenocarcinoma. Furthermore, systemic administration of bevacizumab was chosen over intravitreal injections due to its greater potential to produce effective chemotherapy concentrations around the metastasis via the rich choroidal blood supply, which is within the systemic circulation and is not protected by the blood-retina barrier.

Although we believe that adding bevacizumab helped in achieving complete remission in this patient, some cases were reported in the literature where patients had remission of choroidal metastases from a lung primary cancer after chemotherapy alone or observation. Shah et al. described 22 patients with lung cancer treated with chemotherapy alone with 68% regression of choroidal metastasis and 15 eyes managed with observation alone with 20% regression [16]. Radiation therapy was given as a consolidation treatment because we aimed to cure this patient, where choroidal metastasis was the only extrapulmonary lesion. This patient was treated as having oligometastatic lung cancer, where guidelines recommend that the unique metastasis should be removed surgically or with radiation after chemotherapy, in order to cure the patient [17, 18].

One similar case was previously described in the literature by George et al. [12], showing complete remission of choroidal metastases of poorly differentiated large cell carcinoma after chemotherapy with gemcitabine and carboplatin and systemic bevacizumab. To the best of our knowledge, this is the first case of choroidal metastasis from pulmonary adenocarcinoma, which was successfully treated by chemotherapy and systemic bevacizumab, without any ocular or systemic toxicity.

Further studies are warranted to confirm safety, efficacy, and optimal schedule of such treatment modality and to show the benefit of adding bevacizumab to chemotherapy in patients having choroidal metastases.
